# Daily physical activity and cardiorespiratory fitness in adults with Down syndrome with and without congenital heart disease

**DOI:** 10.1016/j.dhjo.2025.101778

**Published:** 2025-01-28

**Authors:** Julianne G. Clina, David A. White, Joseph R. Sherman, Jessica C. Danon, Daniel E. Forsha, Brian C. Helsel, Richard A. Washburn, Joseph E. Donnelly, Lauren T. Ptomey

**Affiliations:** a Department of Internal Medicine, University of Kansas Medical Center, 3901 Rainbow Boulevard, Kansas City, KS, 66160, USA; b Ward Family Heart Center, Children’s Mercy Kansas City, Kansas City, MO, 64108, USA; c Department of Neurology, The University of Kansas Medical Center, 3901 Rainbow Boulevard, Kansas City, KS, 66160, USA

**Keywords:** Down syndrome, Physical activity, Congenital heart disease

## Abstract

**Background:**

Individuals with Down syndrome (DS) typically exhibit lower cardiorespiratory fitness and reduced moderate-to-vigorous physical activity (MVPA) compared to persons without disability. Approximately 50–55 % of individuals with DS have congenital heart disease (CHD), which is associated with cardiopulmonary deficiencies and reduced MVPA participation in non-DS populations. It is unknown if CHD related comorbidities compound with DS associated deficits in physical activity and fitness.

**Objective:**

To compare physical activity, cardiorespiratory fitness, and cardiovascular function, of persons with DS with and without CHD.

**Methods:**

Baseline data were used from a 12-month randomized controlled physical activity intervention of adults with DS. Participants with DS were age and sex matched based on presence of CHD. Measures of physical activity through accelerometry (n = 42; CHD, n = 21), cardiorespiratory fitness (VO_2peak_; n = 34, CHD n = 17), and cardiovascular function (anaerobic threshold, chronotropic index, O_2_ pulse; n = 34, CHD n = 17) were compared by CHD status using Wilcoxon rank sum tests.

**Results:**

There were no differences in VO_2peak_ between those with and without CHD (CHD 20.3 ml/kg/min; no CHD 21.3 ml/kg/min, *p* = 0.44). MVPA was lower for those with CHD vs. without CHD (10.0 vs 13.3 min/week, *p* = 0.05). There were no differences in cardiovascular function by group.

**Conclusion:**

Fitness and physical activity were low regardless of CHD status. Adults with DS and CHD may engage in less physical activity than those without CHD, however fitness and cardiovascular function were not further impaired by CHD. Given the prevalence of CHD in DS, it is important to include those with CHD in work increasing physical activity and fitness.

## 1. Introduction

Trisomy 21, or Down Syndrome (DS), is the most common genetic cause of intellectual disability with an incidence rate of approximately 1 in 650 live births.^1^ Cardiorespiratory fitness and moderate-to-vigorous physical activity (MVPA) are lower, while sedentary time is higher in adults with DS compared to typically developed adults^2^ and may contribute to an increased risk for obesity, type 2 diabetes, dyslipidemia, dementia and deficits in performing activities of daily living.^3–5^ For these reasons, it is important to identify factors, including the potential impact of pre-existing health conditions, that may contribute to poorer physical activity engagement or fitness to tailor future interventions or identify particularly at-risk targets within the population of persons with DS.

Estimates suggest that approximately 50–55 % of individuals with DS have a congenital heart disease (CHD) diagnosis.^6,7^ High prevalence of CHD in this population is due to the trisomy on chromosome 21 which includes several genes critical to proper heart formation.^8^ Types of CHDs observed in the DS population are often grouped into four categories, listed from most prevalent to least prevalent: 1) Shunt defects (i.e., isolated atrial and ventricular septal defects, and isolated patent ductus arteriosus); 2) Complex defects (i.e., atrioventricular septal defects, aortic arch abnormalities, tetralogy of Fallot, transposition of the great arteries, and single ventricle heart disease); 3) Other defects; and 4) Valve defects (i.e., aortic, pulmonary, and mitral-tricuspid valve defects).^6^

For many CHDs, especially complex defects, surgical repair is necessary within the first five years of life.^9,10^ Unfortunately, CHD related comorbidities still persist across the lifespan, including long-term physiological impairments such as pulmonary deficiencies,^11^ diminished cardiorespiratory fitness,^12^ and impaired gross motor and physical functions.^13^ When CHD related comorbidities are compounded with DS associated deficits in cardiorespiratory fitness, MVPA, and gross motor/physical function, it is unknown if those individuals experience enhanced physiological impairments compared to those with DS without CHD.

The purpose of this cross-sectional analysis was to compare physical activity engagement using accelerometry, cardiorespiratory fitness (VO_2peak_, exercise test time to VO_2peak_, anaerobic threshold), and cardiovascular function (blood pressure, chronotropic index, O_2_ pulse) across age and sex matched adults with DS with and without CHD. Participants or their caregivers self-reported presence of CHD and only those who received surgery for their CHD were included as having a CHD for this analysis. We hypothesized that those with CHD would have poorer physical activity engagement, fitness, and measures of cardiovascular function than those without CHD.

## 2. Methods

This is a cross-sectional analysis using baseline data from a 12-month randomized controlled physical activity intervention for adults with DS.^14^ All data were collected between January 2020 and November 2022. The trial was approved by the University’s Institutional Review Board and registered online. Informed consent and assent were obtained from participants and their parent/legal guardians (if applicable) prior to data collection. Informed consent and assent were obtained from participants and their parent/legal guardians (if applicable) prior to data collection.

### 2.1. Participants

Participants were adults (≥18 years of age) with DS who were living at home with a parent or guardian, or in a supported living environment with a caregiver who agreed to serve as a study partner. Additional inclusion criteria were mild to moderate intellectual disabilities, sufficient functional ability to understand directions, ability to communicate through spoken language, ability to participate in physical activity, walk 10 feet unassisted, and having internet access in the home. Participants were excluded if they had dementia as determined by the Dementia Screening Questionnaire for Individuals with Intellectual Disabilities (DSQIID),^15^ currently participate in a regular exercise program (i.e. ≥20 min/day, ≥3 day/week.) due to the requirements of the parent study, had a serious medical risk, such as cancer or a recent cardiac event as assessed by the primary healthcare provider, or were unable to participate in MVPA. Participants were recruited using flyers, list-serves, and social media posts by local organizations that provide services to adults with DS (e.g. Down syndrome societies, day service programs, and clinics and community developmental disability organizations funded by Medicare).

### 2.2. Assessment of CHD

Participants were assessed for CHD at baseline via health history questionnaire completed by their caregiver or guardian. The questionnaire asked if participants had any history of a CHD (yes/no), what type of CHD they had (open answer), and if they had received a corrective surgery for their CHD (yes/no). Participants were categorized as having a CHD if they reported CHD present at birth and received a corrective surgery. Those with CHD at birth without corrective surgery were not included in this analysis, thus only those who had a CHD that required a corrective surgery were included as having a CHD.

#### 2.2.1. Outcome measures

##### 2.2.1.1. Physical activity.

Sedentary time, light physical activity, and MVPA were assessed using an ActiGraph wGT3XBT tri-axial accelerometer (ActiGraph LLC, Pensacola, FL) worn on the non-dominant hip at the anterior axillary line during waking hours for 7 consecutive days. Vertical axis data aggregated over 60 s epochs from the ActiGraph devices were initialized and downloaded using Actilife software version 6.13.3 (ActiGraph LLC, Pensacola, FL). Non-wear time was defined as at least 90 consecutive minutes of zero counts, with an allowance for 1–2 min of movement between zero and 100 counts/min 16. Counts ≥20,000 min^−1^ were considered spurious.^17^ A wear time ≥8 h on at least 3 days including 1 weekend day was required for inclusion in the analysis. Vertical axis ActiGraph cut points used for adults in the 2003–2004 and 2005–2006 cycles of NHANES were used to classify minutes of sedentary time (<1.0 MET; ≤100 counts/min), light PA (≥1.0 MET, 101–2019 counts per minute), and MVPA (≥3 METs; ≥2020 counts/min).^18,19^ Total PA was calculated by adding light PA and MVPA for each participant.

##### 2.2.1.2. Cardiorespiratory fitness.

VO_2Peak_ measured in ml/kg/min was assessed using a peak treadmill test, following the protocol described by Fernhall et al. developed for adults with DS.^20^ Participants began the test with a 2-min acclimation period to the mask, then the treadmill began at 2 miles per hour at a 0 % incline for 2 min. Every 2 min, incline was increased 2.5 % until 12.5 % include was reached at which point speed was increased by 0.5 miles per hour until criteria for test termination was met. Expired O_2_ and CO_2_ was measured using indirect calorimetry (ParvoMedics TrueOne 2300, Salt Lake City, Utah), which was calibrated with known volume and gas concentrations prior to each test according to manufacturer recommendations. Values were averaged over 15-s intervals across the treadmill protocol. The exercise test was terminated if participants were unable to maintain the treadmill speed or requested that the test be stopped. Otherwise, tests were terminated when participants achieved two or more of the following criteria: 1) volitional exhaustion, 2) a plateau in VO_2_, i.e., <150 ml/min or heart rate (HR) < 2 beats/min with increased work rate, 3) a heart rate within 5 beats/min of peak heart rate predicted using the formula of Fernhall et al.,^20^ and 4) a respiratory exchange ratio ≥1.0. Only participants who achieved a respiratory exchange ratio ≥1.0 or had a heart rate within 10 beats/min of their peak heart rate were included in the analysis. Time to reach VO_2 Peak_ was also recorded for each participant. Ventilatory anaerobic threshold (VAT) was determined by increase by two independent readers by the point when a participant’s ventilation showed a non-linear increase.^21^ Data is presented as the percent of VO_2Peak_ that the VAT was attained.

##### 2.2.1.3. Cardiovascular function.

Resting blood pressure and heart rate were measured on non-dominant arm with participants seated in a chair and both feet flat on the ground. An aerobic step was placed under the feet of those participants who were unable to place their feet on the ground. After 5 min of quiet rest in a chair, blood pressure was taken in duplicate with 2 min separating the first and second measurements. A third measurement was taken for systolic and diastolic values ≥ 5 mmHg apart and the average of the two closest measures was used for analysis. During the cardiorespiratory fitness assessments, heart rate was captured via a five-lead electrocardiogram (CardioCard system, Nasiff associates, Central Square, New York). Chronotropic index is a measure of expected versus achieved maximal heart rate during exercise, with lower values indicating potential cardiovascular impairment during exercise, and was calculated as $\left(\mathrm{Chronotropic index}=\frac{\left(\mathrm{achieved peak HR}-\mathrm{resting HR}\right)}{\left(\mathrm{predicted peak HR}-\mathrm{resting HR}\right)}\right)$ ^22,23^. The value for predicted heart rate peak was calculated using the equation by Fernhall et al.,^20^ of ((*HR* = 210 − 0.56 × age − 31). O_2_ pulse is the product of stroke volume and c(a-v)O_2_ and thus during incremental staged exercise, O_2_ pulse is recognized as a surrogate index of forward stroke volume.^24^ O_2_ pulse is calculated as a ratio between ml of oxygen uptake per heartbeat.

##### 2.2.1.4. Statistical analysis

###### 2.2.1.4.1. Matching.

In order to maximize sample size for physical activity and cardiovascular function and fitness analyses, separate matching was performed for each analysis using case-control matching and performed using SAS (version 9.4, 2002–2012 by SAS Institute Inc., Cary, NC, USA) as described by Mortensen et al.^25^ For the physical activity data, participants with and without CHD with valid accelerometer data were matched 1:1 by sex and age (±3 years). Out of the 81 participants in the parent trial, 67 participants (82.3 %) returned valid data, 23 of which had a CHD and 21 were successfully matched to participants without CHD. For the analysis of fitness related data, participants with and without CHD who completed the treadmill test were matched 1:1 by sex and age (±3 years). Out of the 81 participants in the parent trial, 56 (69.1 %) completed the treadmill test at baseline, 18 of which had a CHD and 17 were successfully matched to participants without CHD.

###### 2.2.1.4.2. Analysis.

All outcome measures were compared by CHD status using non-parametric tests due to non-normal data distribution. Wilcoxon rank sum tests were used to compare all outcomes by CHD status. Only complete pairs were included in the analysis of each specific outcome. Cohen’s d effect sizes were calculated using the mean scores by group and the pooled standard deviations. Effect sizes are interpreted as d = 0.20 (small), d = 0.50 (medium), and d = 0.80 (large).^26^ All analyses were performed using SAS (version 9.4, 2002–2012 by SAS Institute Inc., Cary, NC, USA).

## 3. Results

### 3.1. Physical activity

A total of 42 participants with DS (with CHD n = 21; without CHD n = 21) were successfully matched and included in the analyses for physical activity. Demographics of the sample and CHD and non-CHD groups are displayed in Table 1. The average age of the sample was 25.4 years and 61.9 % of the sample were female (n = 26). Most of the participants were White race (n = 38, 90.5 %) and non-Hispanic ethnicity (n = 37, 88.1 %). Participants had an average BMI of 32.2 ± 1.1, with 54.8 % (n = 23) of the sample having obesity (BMI≥30.0 kg/m^2^). Groups did not differ in any demographic characteristic. Among those with CHD, the most common defect was ventricular septal defect (n = 5, 23.8 %), followed by atrioventricular septal defect (n = 4, 19.0 %). Five participants (23.8 %) reported two or more defects.

Physical activity and sedentary time outcomes can be found in Table 2. Average MVPA was lower in participants with CHD than without CHD (10.0 ± 2.7 min/d vs. 13.3 ± 1.9 min/day, *p* = 0.05) and had a small effect size of 0.31. Light PA and Total PA were also lower in those with CHD, however this difference did not reach statistical significance (*p* = 0.07 and *p* = 0.06 respectively) despite a medium effect size for both variables (Cohen’s d = 0.58 for both).

### 3.2. Cardiorespiratory fitness and function

A total of 17 persons with CHD completed the treadmill test and were successfully matched by age and sex to persons without CHD. Demographics of this sample are presented in Table 3. Average age for this sample was 25.2 years and 70.6 % of the sample were female (n = 24). Most of the participants were White race (n = 30, 88.2 %) and non-Hispanic ethnicity (n = 29, 85.3 %). Participants had an average BMI of 32.5 ± 1.2, with 58.8 % (n = 20) of the sample having obesity (BMI≥30.0 kg/m^2^). Groups did not differ in any demographic characteristic. Among those with CHD, the most common defect was atrioventricular septal defect (n = 6, 35.2 %), followed by ventricular septal defect and patent ductus arteriosus which were present in 2 participants (11.8 %) each. Four participants (23.5 %) reported two or more defects.

Results related to cardiorespiratory fitness and cardiovascular function are presented in Table 4. There was no difference in VO_2Peak_, time to reach VO_2Peak_, or maximal heart rate, between the CHD and non-CHD groups. As observed by others, VAT can be a challenge to detect in some individuals with DS.^27^ VAT was able to be detected in 58.8 % of participants (10 of 17 in the CHD group and 10 of 17 in the no CHD group). There was no difference in percent of VO_2Peak_ that the VAT was attained between CHD and non-CHD groups. Resting diastolic blood pressure was significantly lower in participants without CHD compared to those with CHD (60.8 vs 72.6; *p* = 0.0003). However, there were no differences in resting systolic blood pressure, or chronotropic index between groups. Additionally, there were no differences in resting, peak, or rest-to-peak change in O_2_ pulse between groups.

## 4. Discussion

Previous work has identified that adults with DS have lower fitness and physical activity engagement^2^ than typically developed adults, however the impact of CHD on these factors for adults with DS has been unexplored. This study aimed to evaluate the impact of CHD on fitness, physical activity, and cardiovascular function of adults with DS by comparing matched pairs with and without CHD. The results of this analysis indicate that adults with DS with CHD have lower MVPA, and lower resting diastolic blood pressure compared to those without CHD. However, there were no differences between participants with and without CHD for VO_2Peak_, percent of VO_2Peak_ that the VAT was attained, step counts, sedentary time, light physical activity minutes, systolic blood pressure, chronotropic index, and metrics of O_2_ pulse.

Individuals with and without CHD had similar VO_2Peak_ and VAT indicating that presence of a CHD might not be the primary driver of impaired exercise capacity in adulthood for this population, in the same way it does in individuals without DS.^28^ Both groups with and without CHD had lower VO_2Peak_ than is seen in the general population, which aligns with previous research findings that cardiorespiratory fitness is lower for adults with DS.^12^ Furthermore, the VO_2Peak_ values seen in this study are similar to other literature assessing VO_2Peak_ in adult populations with DS.^29,30^ The similarities in exercise capacity in our study and previous research in non-CHD adults with DS provide further evidence that the primary limitations in exercise capacity may be moderated by presence of DS instead of CHD status. Still, this should be investigated further as the sample analyzed in this paper included only 17 matched pairs.

Those with CHD had significantly lower MVPA than those without (10.0 vs 13.3 min per day), however overall physical activity engagement was low in both groups. Neither group met the current 150 min per week guideline of moderate activity.^31^ Additionally, both groups exceeded 8 h of sedentary time per day. This is in line with current literature suggesting that those with DS have a low level of physical activity.^2^ While time spent in light physical activity did not differ statistically, those with CHD reported approximately 50 min less of light physical activity per day. Findings from this analysis suggest that those who have a CHD and DS might engage in less PA than those without CHD which should be further investigated in future work with a larger sample. Previous research suggest that adults without DS participate in 20–40 min of MVPA per day.^32^ In contrast, our sample exhibited lower MVPA engagement, regardless of CHD status. It is well-documented that individuals with DS generally engage in less physical activity compared to their peers without DS^12^ often due to physiological factors (e.g., lower fitness levels, gait instability) and environmental barriers (e.g., limited access to fitness programs, transportation challenges). However, this analysis did not include a control group of adults without DS for comparison, a limitation stemming from the design of the parent study, which focused exclusively on an exercise intervention for adults with DS. Future research should address this gap by incorporating a control group to better contextualize these differences.

In those with CHD without DS, MVPA engagement is often reported to be lower than in those without CHD.^33^ Common barriers to physical activity in this population include perceived safety concerns related to their heart condition, with many parents expressing fear about their child with CHD participating in physical activity.^34^ Similarly, qualitative research has highlighted that many adults with CHD fear that physical activity could worsen their heart condition or overall health, further hindering participation.^34^ Additionally, individuals with CHD are frequently advised by physicians to limit exercise, even in cases of mild CHD, which may amplify fears and contribute to reduced physical activity levels.^33^ These factors may help explain the lower physical activity engagement observed in this analysis. Fear of exercise in individuals with both DS and CHD should be explored further. Moreover, research is needed to assess the safety and efficacy of physical activity interventions for individuals with DS and CHD. Such work could expand understanding of exercise safety in this population, inform clinical care practices, and improve CHD education and management strategies.

Interestingly, we did not observe differences in exercise-based measures of cardiovascular function between CHD and non-CHD groups. It is known that adults with DS have both impairments in cardiorespiratory fitness and lower maximal heart rate^12^ which are thought to related to autonomic dysfunction,^35^ and may result in lower cardiac output and exercise capacity.^36^ Poor chronotropic index is a well-documented predictor of mortality among individuals with and without known or suspected cardiovascular disease.^37^ O_2_ pulse, a surrogate of forward stroke volume – a major contributor to cardiac output, has been identified as a predictor for coronary artery disease and mortality in healthy adults.^38^ In adults with CHD, O_2_ pulse is associated with left ventricular stroke volume, systolic blood pressure, and VO_2peak_.^39^ In contrast to our findings, research in non-DS individuals observed that those with CHD had O_2_ pulse values that were 2.4–3.1 ml/beat lower than controls without CHD.^40^ Together, these findings suggest that it is possible that presence of CHD may not be adding greater risk for the development of cardiovascular disease than that already present in individuals with DS. However future work should investigate this in a larger sample to draw stronger conclusions.

While CHD is exceptionally common among populations with DS, little research has investigated the effect of severe CHD on fitness or physical activity engagement. With over 50 % of those with DS having a significant CHD,^6^ it is important to include this group in research related to exercise. People with DS commonly report lower physical activity levels than their typically developing peers^2^ and data from this study suggest that those with DS and CHD might be participating in less physical activity than those with DS without CHD. This group could be an important intervention target for future research. Additionally, it is important to investigate the effect of exercise interventions on people with DS with and without CHD to determine if such interventions are effective across groups for increasing physical activity engagement and fitness. As this was a cross-sectional analysis of baseline data, the safety and effectiveness of the intervention for individuals with versus without CHD were not evaluated. This represents a critical next step to determine whether exercise can be implemented safely and effectively in this population. Individuals with CHD often experience heightened concerns and fear regarding exercise, which may necessitate modified or supplementary intervention materials to appropriately promote increased physical activity and fitness. Future interventions should also consider incorporating additional education and resources tailored to individuals with CHD to address their specific fears and barriers to exercise. Such enhancements could help alleviate concerns and improve engagement, ultimately supporting more effective intervention outcomes for this population.

Limitations of this work include a small sample size and a relatively homogenous sample in terms of race and ethnicity, with an average age of ~27 years, and only included sedentary adults with DS, as was required for the parent study. Thus, future research should work to include adults with DS from various racial and ethnic backgrounds, age groups, and physical activity engagement categories in order to understand how these factors may impact fitness and cardiovascular function. Incorporating a comparison group of adults without DS could also enhance future studies, particularly if individuals with CHD but without DS are included. Additionally, this study did not collect information on income or education levels of individuals with DS or their families, which could potentially influence the outcomes examined in this analysis. Including these variables in future research could help identify whether socioeconomic factors impact the observed results. While data on the type of CHD was collected, the sample size was too small to explore how different CHD types may uniquely affect physical activity engagement and fitness. Expanding the sample size in future studies would allow for such analyses and provide valuable insights into the nuanced effects of CHD in this population.

## 5. Conclusion

People with DS with and without CHD report low levels of physical activity and poor fitness. Individuals with CHD may be at higher risk lower physical activity engagement than those without CHD. Interventions designed to increase physical activity are important for improving health and wellbeing of people with DS and should include those with CHD.

## Figures and Tables

**Table 1:** Accelerometer data demographics.

Outcome	Total (n = 42)	CHD (n = 21)	No CHD (n = 21)
Age, y (M±SE)	25.4 ± 1.1	25.2 ± 1.6	25.7 ± 1.5
Female, n (%)	26 (61.9)	13 (61.9)	13 (61.9)
Weight, kg (M±SE)	72.5 ± 2.6	73.7 ± 3.5	71.3 ± 3.8
BMI (kg/m^2^) (M±SE)	32.2 ± 1.1	32.4 ± 1.6	32.1 ± 1.5
BMI≤25 kg/m^2^	6 (14.3)	3 (14.3)	3 (14.3)
BMI >25, <30 kg/m^2^	13 (31.0)	7 (33.3)	6 (28.6)
BMI≥30 kg/m^2^	23 (54.8)	11 (52.4)	12 (57.1)
White, n (%)	38 (90.5)	19 (90.5)	19 (90.5)
Hispanic or Latino, n (%)	5 (11.9)	2 (9.5)	3 (14.3)
Heart condition, n (%)			
ASVD		4 (19.0)	
ASD		3 (14.3)	
VSD		5 (23.8)	
PDA		2 (9.5)	
Two or more		5 (23.8)	
Other		2 (9.5)	

Abbreviations: ASD, atrial septal defect; ASVD, atrioventricular septal defect; BMI, body mass index; kg, kilogram; m, meter; M, mean; PDA, Patent ductus arteriosus; SE, standard error; VSD; ventricular septal defect; y, year.

**Table 2:** Accelerometer outcomes by CHD status.

Outcome	CHD (n = 21)	No CHD (n = 21)	*p* value	*% difference*	Cohen’s d
Steps	4152.6 ± 382.0	5051.9 ± 503.7	0.19	19.5	0.44
Sedentary Time (min/d)	492.0 ± 23.7	473.8 ± 38.1	0.32	3.8	0.13
Light PA (min/d)	251.9 ± 16.5	302.6 ± 21.1	0.07	18.3	0.58
MVPA (min/d)	10.0 ± 2.7	13.3 ± 1.9	0.05	28.3	0.31
Total PA (min/d)	261.9 ± 17.6	316.0 ± 22.6	0.06	18.7	0.58

Note: Data presented as mean ± standard error. Differences by group compared using Wilcoxon rank sum tests.

Abbreviations: CHD, congenital heart defect; d, day; min, minutes.

**Table 3:** Fitness data demographics.

Outcome	Total (n = 34)	CHD (n = 17)	No CHD (n = 17)
Age, y (M±SE)	25.2 ± 1.2	25.1 ± 1.7	25.2 ± 1.7
Female, n (%)	24 (70.6)	12 (70.6)	12 (70.6)
Weight, kg (M±SE)	72.6 ± 3.1	73.0 ± 4.5	72.2 ± 4.5
BMI (kg/m^2^) (M±SE)	32.5 ± 1.2	32.4 ± 1.7	32.5 ± 1.7
BMI≤25 kg/m^2^	4 (11.8)	2 (11.8)	2 (11.8)
BMI >25, <30 kg/m^2^	10 (29.4)	6 (35.2)	4 (23.5)
BMI≥30 kg/m^2^	20 (28.8)	9 (52.9)	11 (64.7)
White, n (%)	30 (88.2)	16 (94.1)	14 (82.4)
Hispanic or Latino, n (%)	5 (14.7)	3 (17.6)	2 (11.8)
Heart condition, n (%)			
ASVD		6 (35.2)	
ASD		1 (5.9)	
VSD		2 (11.8)	
PDA		2 (11.8)	
Two or more		4 (23.5)	
Other		2 (11.8)	

Abbreviations: ASD, atrial septal defect; ASVD, atrioventricular septal defect; BMI, body mass index; kg, kilogram; m, meter; M, mean; PDA, Patent ductus arteriosus; SE, standard error; VSD; ventricular septal defect; y, year.

**Table 4:** Cardiorespiratory fitness outcomes by CHD status.

Outcome	CHD (n = 17)	No CHD (n = 17)	*p* value	*% difference*	*Effect Size*
VO_2Peak_ (ml/kg/min)	20.3 ± 1.0	21.3 ± 0.9	0.44	4.8	0.25
Time to VO_2Peak_ (seconds)	653.0 ± 3.6	708.9 ± 42.5	0.07	8.2	0.49
Maximal Heart Rate (bpm)	156.0 ± 2.9	163.5 ± 2.7	0.10	4.7	0.64
Systolic Blood Pressure	117.3 ± 3.2	113.3 ± 3.0	0.26	3.5	0.32
Diastolic Blood Pressure	60.8 ± 2.0	72.6 ± 1.8	0.0003	17.7	6.16
VAT	76.3 ± 5.8	84.1 ± 4.1	0.33	9.7	0.49
Peak O_2_ Pulse	9.1 ± 0.6	9.5 ± 0.6	0.73	4.3	0.17
Resting O_2_ Pulse	2.0 ± 0.2	2.0 ± 0.2	0.90	0.0	0.00
O_2_ Pulse Difference	8.0 ± 0.4	7.7 ± 0.5	0.42	3.8	0.21
Chronotropic Index %	90.1 ± 3.7	98.2 ± 3.1	0.12	8.6	0.57

Note: Data presented as mean ± standard error. Differences by group compared using Wilcoxon rank sum tests. Sample size was 20 for VAT variable (CHD n = 10; no CHD n =10).

Abbreviations: bpm, beats per minute; CHD, congenital heart defect; Hg, mercury; kg, kilogram; min, minute; ml, milliliter; mm, millimeter; VAT, Ventilatory anaerobic threshold.

## Data Availability

Deidentified individual participant data (including data dictionaries) will be made available, in addition to study protocols, the statistical analysis plan, and the informed consent form. The data will be made available upon publication to researchers who provide a methodologically sound proposal for use in achieving the goals of the approved proposal. Proposals should be submitted to the corresponding author at jclina@kumc.edu.
